# The Effects of a Computer Game (Healthy Rat King) on Preschool Children’s Nutritional Knowledge and Junk Food Intake Behavior: Nonrandomized Controlled Trial

**DOI:** 10.2196/33137

**Published:** 2022-07-01

**Authors:** Ing-Chau Chang, Cheng-Ying Yang, Chin-En Yen

**Affiliations:** 1 Department of Computer Science and Information Engineering National Changhua University of Education Changhua Taiwan; 2 Department of Computer Science University of Taipei Taipei Taiwan; 3 Department of Early Childhood Development and Education Chaoyang University of Technology Taichung Taiwan

**Keywords:** computer games, nutrition knowledge, junk food

## Abstract

**Background:**

Playing computer-aided games could enhance children’s interest in learning about nutritional knowledge and eventually promote healthy dietary intake behavior.

**Objective:**

This study aims to evaluate the effectiveness of a computer game (Healthy Rat King) in improving the knowledge on nutrition and junk food intake among preschool children in Taiwan.

**Methods:**

This was a quasi-experimental study that utilized the computer game Healthy Rat King as the nutrition education tool. We recruited 104 preschool children (aged 5-6 years) from preschools in central Taiwan, who were assigned to either the experimental group (n=56) or the control group (n=48). In the experimental group, a 1-hour computer-based educational game intervention was included in the course for 4 consecutive weeks. The control group did not receive this intervention.

**Results:**

The level of nutritional knowledge for children in the experimental group was significantly higher than those in the control group after 4 weeks (*P*=.002). Furthermore, the frequency of consumption of chocolate, candies, and ice cream (high-calorie junk food) was reduced in the experimental group. There was also no significant difference in the consumption of candy and chocolate (*P*=.54), ice cream and ice pops (*P*=.21), cake (*P*=.92), biscuit (*P*=.98), soft drinks (*P*=.52), and fruit juice and sugary drinks (*P*=.31) between the 2 groups in the posttest.

**Conclusions:**

Teaching using a computer game could improve children’s nutritional knowledge. However, the intake frequency of junk food among children in the experimental group showed no significant difference from those in the control group.

## Introduction

Recently, nutrition-related problems in children, such as obesity, unbalanced diet, and high intake of junk food, have been an area of extensive focus among parents, child nutrition experts, and teachers on early childhood education. It has been reported that 93% of children intake packaged food, 68% consume sugary drinks more than once a week, and 53% intake foods that are high in fat, salt, calories, or sugar at least once a day [1]. Besides, a previous survey [2] showed that 21% of children in Taiwan drink sugary beverage at least once a day, while 89% of children eat junk food.

The preschool age (ie, age 3-6 years) is an important period to set or correct the eating habits of children. The formation of eating habits not only impacts the individual’s food choices but also incurs the health, learning, and behavior problems [3]. Studies have shown that young children with malnutrition might have lower cognitive function scores, poor psychological development, reduced fine motor skills, and limited activity [3,4]. Importantly, the eating behavior (eg, food choices or motives) will impact the occurrence of diseases in the future. “Junk food” is unhealthy, as it is high in calories, fat, sugar, and salt. Further, it will increase the risk of malnutrition, obesity, cardiovascular diseases, high blood pressure, diabetes, and other chronic diseases [5]. Its regular consumption might also result in lower vitamins, minerals, and essential fatty acids that eventually affect the growth and development of children [3]. Therefore, it is very important to establish correct eating habits in the early childhood period.

For young children, nutrition education is an important tool in the preschool period. Implementation of nutrition education in preschools could well improve nutritional knowledge and eating habits of young children. Teachers in preschools conduct nutrition education sessions according to the interests and development of young children, and increase the participation and experience of young children in the teaching process [6].

Computer represents an important tool for learning. Computer games are novel and attractive to children. They gradually become their favorite because of their simple, convenient, vivid, interesting, entertaining, and challenging features [7]. Therefore, applying computer games as a teaching tool could help in the development of children’s cognitive abilities, interactive relationships, and operational skills [8]. As computer games combine entertainment with knowledge, the concept of learning while playing might achieve the purpose of education. Besides, a computer game could help with behavior development, character building, and skill development for children [8].

Many studies have used computer games to understand the relationship between children’s nutritional knowledge and eating behaviors [9-11]. Results of these studies have shown that computer games have a positive impact on children’s dietary intake, nutritional knowledge, attitudes, behaviors, and activity level [9-11]. Several studies indicate that after playing video games for 1-6 weeks, the children have a positive attitude toward healthy eating, make healthy food choices, and reduce their sugar consumption; additionally, their attitudes on nutrition and physical activity improved. These results support the use of educational computer games as viable tools to help young children improve their food knowledge and dietary behaviors [10,11].

However, some results show that computer games could not change children’s long-term eating behaviors. Although a short-term computer game intervention might improve children’s nutritional knowledge, it does not sufficiently impact their nutritional knowledge and actual eating behavior in the long term [9].

Thus, computer games could have positive effects on nutritional knowledge, dietary intake, attitudes, and behaviors of children [9,12]. Computer game is also an appropriate nutrition education tool to improve children’s attitudes toward food. One limitation of previous studies (eg, [9,11]) was that they primarily included children aged 8-13. As a result, less studies are available for preschool children. Nowadays, computer games are rarely used as a teaching tool in the preschools. We thus performed this study to evaluate whether computer games could enhance children’s learning motivation, computer games included in teaching activities are effective, and computer game–related product development are appropriate for preschool teachers. We will also evaluate the effects of educational games on children’s nutritional knowledge and junk food intake behaviors.

## Methods

### Participants

This was a quasi-experimental study. We recruited 104 preschool children (aged 5-6 years) from preschools in central Taiwan, who were assigned to either the experimental group (n=56) or the control group (n=48). In the experimental group, a 1-hour computer-based educational game intervention was included in the course for 4 consecutive weeks. The control group did not receive this intervention. Informed consent was obtained from the parents of the participating children.

### Computer Game Design

For the Construct 2 course, an HTML5-based computer game (Healthy Rat King) was implemented as the auxiliary nutrition education tool for children in the experimental group. After installing the necessary software, the teacher just clicked the file named *index.html*, executed the computer game program, and then used the game to teach Construct 2.

### Background of the Healthy Rat King Game

The Healthy Rat King game involves a situation in which a mouse hides from cats in the forest, while various foods appeared along the escape route to replenish its energy. If the mouse ate healthy foods, it earned 10 points and ran faster. By contrast, if it ate junk foods, it lost 5 points, gained “unhealthy” weight, and ran slower. The game gets over if the mouse is caught by the cat (usually with a low score) or if it reaches home without being caught (usually with a high score).

The goal of Healthy Rat King is to make young children understand foods that are healthy, while it also educates them about foods with high fat, high sugar, and high calorie content (junk food). Children can learn an important concept from this game: eating too much junk food can easily make one both fat and unhealthy. Thus, people should avoid eating too much junk food, and instead consume healthier food options.

Before performing the formal study, 12 children were selected for a pretest to evaluate the suitability of the computer game.

### Learning Sheet

The nutritional knowledge status of all children was evaluated using the learning sheet before and 4 weeks after the intervention. This learning sheet was designed by the researcher (C-YY) and verified by 3 experts according to the purpose of the research, the appropriateness of the content of the inspection topic, and the clarity of the questionnaire. The learning sheet includes 3 major questions. The first and second questions were used to examine the children’s cognition on unhealthy and healthy food choices (ie, what are junk foods and healthy foods). The third question was used to examine children’s cognition on the influence of junk food intake on the body (ie, the effects of eating junk food on the body, such as easy to getting sick or obese). The full score on the learning sheet is 100 points.

### Questionnaire

The questionnaire contains the background information (including age, gender, parental education level, parental occupation), dietary surveys, status of junk food intake frequency among preschool children, and the influence of computer game interventions on preschool children’s junk food intake (parent perceptions of the computer game teaching). The questionnaire was filled out by the parents of the preschool children. The reliability of the questionnaire is .90 (Cronbach α). The content validity of the questionnaire was reviewed by 3 experts.

### Study Process

The experimental group received the computer game education for 1 hour once a week for 4 consecutive weeks. The content of the computer game education comprised 3 parts: children’s learning motivation (eg, picture book performance, music rhythm, finger ballad), development activities (computer game learning), and integration activities (group discussion).

In the first stage, a pretest of the computer game is conducted to check teaching contents in 2 preschool classes.

In the second stage, all participating children have to complete the pretest of the learning sheet before the computer game course intervention.

In the third stage, the researchers conduct computer game education courses for the experimental group once an hour every week for 4 consecutive weeks. Each course content included the derived motivation, development activity (playing the computer game), and group discussion. Children in the experimental group were divided into subgroups (4-6 children per subgroup). They take turns to play computer games in the classroom. The playing game status is recorded by researchers. By contrast, the children in the control group did not receive the computer game course intervention. They performed general theme curriculum activities (derived motivation, art or music, or physical development activity) and participated in group discussions.

**Figure 1:**
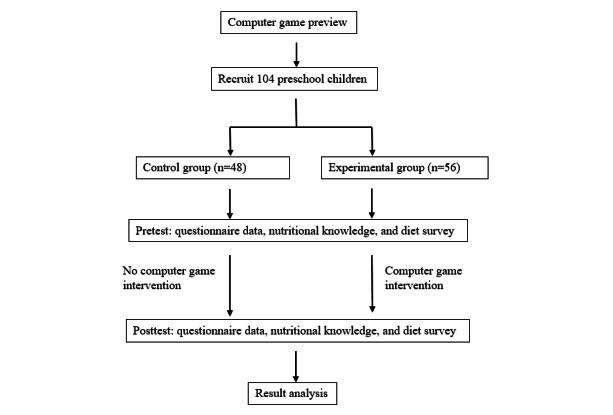
Study flowchart.

In the fourth stage, all participating children have to complete the posttest of the learning sheet after the computer game course intervention. The diet surveys and the junk food intake frequency questionnaire were also filled out by the parents of participating children. The study’s flow diagram is illustrated in Figure 1.

### Statistical Analysis

This study used SPSS for Windows (version 20.0; IBM, Inc.) for data analysis and statistics. Background data were subjected to descriptive statistics. The normality distributions of the data were analyzed by the Kolmogorov-Smirnov test, while a paired sample *t* test was used to compare the difference in nutritional knowledge scores and computer game scores between the pre- and posttest in the experimental group. It was also applied to compare the difference in nutritional knowledge scores between the pre- and posttest in the control group. The frequency of junk food intake among preschool children was described with the Bowker test of symmetry to compare the difference between the pre- and posttest. Differences in the frequency of junk food intake among preschool children between the experimental and control groups were determined using the chi-square test. The independent sample *t* test was used to compare the difference in nutritional knowledge scores between children in the experimental and control groups. A linear regression analysis was used to assess the relationship between the intervention (computer game course) and nutritional knowledge score after controlling for confounding factors (age, gender, parents education level, and parents occupation). A significance level of *P*<.05 was used for all tests.

### Ethical Considerations

 This study was exempt from research ethics review since it taught skills, and the effectiveness evaluation was conducted in a general teaching environment [13].

## Results

Table 1 presents the demographic characteristics of the participants. Overall, there were more children with second birth ranking. Most fathers and mothers had a college degree.

Table 2 shows the computer game scores earned by the preschool children. The mean score was 25 (SD 23), 30 (SD 29), 38 (SD 33), and 37 (SD 25) in the first, second, third, and fourth weeks, respectively. Compared with the first week, a significant increase on game scores was noted in the third (*P*=.02) and fourth (*P*<.001) weeks. About 75% (42/56) of preschool children liked the computer game course *very much*, 13% (7/56) *liked* computer games, and only 5% (3/56) *disliked* the games.

Table 3 presents the nutritional knowledge scores of the preschool children. We used a learning sheet to evaluate the nutritional knowledge of preschool children. The mean pre- and posttest scores of the experimental group were significantly higher than those in the control group (*P*=.26 and <.001 for pre- and posttest, respectively). Furthermore, nutritional knowledge scores of the experimental group were significantly higher than those of the control group in the posttest (*P*=.002).

Table 4 shows the frequency of junk food intake between the experimental and control groups. Junk foods such as candies, soft drinks, cookies, cakes, packed snacks are unhealthy, as they are high in calories, sugar, and fat, but have little dietary fiber, protein, vitamin, minerals, or other important nutritional elements. There was no significant difference in the frequency of consumption of candy and chocolate (*P*=.50), ice cream and ice pops (*P*=.48), cake (*P*=.60), biscuit (*P*=.72), and soft drinks intake (*P*=.70) between the experimental and control groups; however, consumption of fruit juice and sugary drinks showed a significant difference between the experimental and control groups in the pretest (*P*=.02). There was also no significant difference in the consumption of candy and chocolate (*P*=.54), ice cream and ice pops (*P*=.21), cake (*P*=.92), biscuit (*P*=.98), soft drinks (*P*=.52), and fruit juice and sugary drinks (*P*=.31) between the 2 groups in the posttest.

Table 5 presents the relationship between the computer game course intervention and the nutritional knowledge score. The computer games intervention was significantly related to the improvement of children’s nutritional knowledge (*P*=.001).

Following the intervention, the percentage of children who refused to eat junk food was significantly higher than those in the pretest (*P*=.01); additionally, the percentage of parents encouraging junk food as a reward was significantly lower than the pretest (*P*<.001). Most parents (41/56, 73%) agreed that the computer game can improve their children’s nutritional knowledge, reduce the frequency of junk food intake, and improve dietary behavior. Importantly, the intake frequencies of candy and chocolate as well as ice cream and ice pops decreased in the experimental group after the computer game intervention. The top 3 junk foods among the participating children were biscuits, candy and chocolates, and juice and sweetened beverages (data not shown).

**Table 1:** Descriptive characteristics of participants.

Variable and item		All (n=104)	Experimental group (n=56)	Control group (n=48)	*P* value
Age (years), mean (SD)		5.14 (0.27)^a^	5.13 (0.31)	5.15 (0.23)	.67
**Gender, n (%)**					.38
	Male	59 (56.7)	34 (60.7)	25 (52.1)	
	Female	45 (43.3)	22 (39.3)	23 (47.9)	
**Birth ranking, n (%)**					.33
	First	43 (41.3)	22 (39.3)	21 (43.8)	
	Second	50 (48.1)	30 (53.6)	20 (41.7)	
	Third	11 (10.6)	4 (7.1)	7 (14.6)	
**Educational degree of the father, n/N (%)^a^**					.32
	High school or below	46/102 (45.1)	29/55 (52.7)	17/47 (36.2)	
	College	50/102 (49.0)	23/55 (41.8)	27/47 (57.4)	
	Graduate school or above	6/102 (5.9)	3/55 (5.5)	3/47 (6.4)	
**Educational degree of the mother, n/N (%)^a^**					.14
	High school or below	39/103 (37.9)^a^	24/55 (43.6)	15/48 (31.3)	
	College	58/103 (56.3)	30/55 (54.5)	28/48 (58.3)	
	Graduate school or above	6/103 (5.8)	1/55 (1.8)	5/48 (10.4)	

^a^Some participants are single parents.

**Table 2:** The computer game scores of the preschool children.

Variable	Score	*t* value (*df*)	*P* value
First week, mean (SD)	25 (23)	N/A^a^	N/A
Second week, mean (SD)	30 (29)	–1.11 (55)	.27
Third week, mean (SD)	38 (33)^b^	–2.4 (55)	.02
Fourth week, mean (SD)	37 (25)^c^	–2.98 (55)	<.001

^a^N/A: not applicable.

^b^Third week > first week.

^c^Fourth week > first week.

**Table 3:** The scores of nutritional knowledge.

Variable	All	Experimental group (n=56)	Control group (n=48)	*P* value
Pretest, mean (SD)	80 (15)	81 (16)	78 (14)	.26
Posttest, mean (SD)	85 (14)	89 (10)	80 (15)	<.001
Posttest-pretest, mean (SD)	5 (12)	8 (12)	1 (10)	.002

**Table 4:** Frequency of junk food intake between the experimental (n=56) and control (n=48) groups.

Variable and group			<1 time/month, n (%)	1-3 times/month, n (%)	>1 time/week, n (%)	*P* value
**Pretest**						
	**Candy and chocolate**					.50
		Experimental	16 (29)	19 (34)	21 (38)	
		Control	9 (19)	19 (40)	20 (42)	
	**Ice cream and ice pops**					.48
		Experimental	22 (39)	25 (45)	9 (16)	
		Control	14 (29)	23 (48)	11 (23)	
	**Cake**					.60
		Experimental	33 (59)	21 (38)	2 (4)	
		Control	24 (50)	21 (44)	3 (6)	
	**Biscuit**					.72
		Experimental	6 (11)	21 (38)	29 (52)	
		Control	3 (6)	19 (40)	26 (54)	
	**Soft drinks**					.70
		Experimental	36 (64)	15 (27)	5 (9)	
		Control	27 (56)	16 (33)	5 (10)	
	**Fruit juice and sugary drinks**					.02^a^
		Experimental	18 (32)	18 (32)	20 (36)	
		Control	12 (25)	28 (58)	8 (17)	
**Posttest**						
	**Candy and chocolate**					.54
		Experimental	20 (36)	21 (38)	15 (27)	
		Control	13 (27)	18 (38)	17 (35)	
	**Ice cream and ice pops**					.21
		Experimental	31 (55)	21 (38)	4 (7)	
		Control	20 (42)	20 (42)	8 (17)	
	**Cake**					.92
		Experimental	36 (64)	17 (30)	5 (9)	
		Control	29 (60)	16 (33)	3 (6)	
	**Biscuit**					.98
		Experimental	7 (13)	20 (36)	29 (52)	
		Control	6 (13)	18 (38)	24 (50)	
	**Soft drinks**					.52
		Experimental	36 (64)	13 (23)	7 (13)	
		Control	27 (56)	16 (33)	5 (10)	
	**Fruit juice and sugary drinks**					.31
		Experimental	22 (39)	22 (39)	12 (21)	
		Control	15 (31)	26 (54)	7 (15)	

^a^*P*<.05.

**Table 5:** The relationship between computer game intervention and nutritional knowledge score.

Variable	Coefficients	SE	95% CI	*P* value^a^
Nutritional knowledge score^b^	8.45	2.38	3.72-13.19	.001

^a^Adjustment for age, gender, parents education level, and parents’ occupation in the linear regression.

^b^Posttest-pretest score.

## Discussion

### Principal Findings

Our results show that children’s nutritional knowledge scores in the posttest were significantly higher than those in the pretest within the experimental group, whereas the scores of the control group were not significantly different between the pre- and posttest. This shows that Healthy Rat King, an educational game, improves the nutritional knowledge of children. This result is consistent with findings from other studies that computer games could help children correctly distinguish between healthy and unhealthy foods, while also improving the nutritional knowledge of children [9,11,14,15]. The sound, light, audio, and visual effects of computer games could arouse learning motivation and interest among children. By integrating computer games into education, children can maintain their enthusiasm to learn. Computer games have been one of the important methods to arouse active learning motivation and encourage enthusiasm among learners [16].

There was no significant difference in junk food intake between the experimental and control groups in the posttest. However, among children in the experimental group, intake frequencies of both chocolate and ice cream and ice pops decreased after the intervention. Children preferred to eat healthy foods after playing the computer game, Healthy Rat King, suggesting positive changes in attitudes and behaviors. This result is similar to that of previous studies in which school-aged children who received a computer game education increased their consumption of vegetables, fruits, and other healthy foods, and reduced junk foods such as French fries, candy, chocolate, cakes, soft drinks, fried foods, and sugary drinks, compared with children who did not receive the computer game intervention [9-11,17,18]. The results of these studies clearly indicate that the application of computer games to nutrition education can effectively improve the eating habits and promote healthy behaviors among young children.

In the experimental group, the percentage of children who refused to consume junk food was significantly higher than that in the pretest, while parents’ use of junk food as a reward for children also decreased after the computer game intervention; however, there was no significant difference in the control group. This result shows that parents have made also some changes (reduction in the provision of junk food as a reward) in their children’s diet based on their children’s opinions (reject junk food). Young children’s eating behavior is affected by many factors, including physiological, experience, learning, and social factors. Among these factors, parent’s eating behavior and family dietary habits are the key to cultivating good eating habits and behaviors among preschool children. If the parents have a high intake of junk food or frequently offer junk foods to their children, then their children will also have these poor eating habits. Therefore, parental eating habits or family food provision has a significant influence on preschool children’s nutritional knowledge and eating behavior [11,19]. Although intake frequencies of candy and chocolate as well as ice cream and popsicles have decreased following the computer game intervention in the experimental group, there was no significant difference in junk food eating behavior between the groups in the posttest. To the best of our knowledge, the family’s eating habits directly impact those of children, and so they need a long time to develop their own eating habits. However, the computer game intervention period in this study lasted for only 4 weeks, which may explain the absence of significant difference in the junk food intake frequencies between these 2 groups in the posttest.

Studies have suggested that computer games can improve children’s nutritional knowledge attitudes and behaviors. However, computer games have some inherent problems. Playing computer games for a long time could easily cause eye fatigue, which leads to a decreased vision. Early childhood is an important stage for eye development, so children should avoid eye damage due to use the excessive use of computer for a long time. Although computer games enrich learning, it could also increase certain injuries [20]. For example, computer games may separate young children from useful gaming experiences (physical activity), reduce opportunities for developing social skills, and cause problems such as lack of social interaction. In addition, unhealthy types of computer games or game plots might be harmful to young children’s physical and mental development [20].

The results of this study show that the application of computer games in nutrition education for preschool children can effectively promote nutritional knowledge. This study can provide inspiration for the design of computer games and teaching tools suitable for preschool children.

### Limitations

The computer game, Healthy Rat King, designed for the purpose of nutrition education is simple, cute, interesting, and fun. Although the game meets the psychological needs of children, some children face difficulties in using computers, which might lead to a bias in the computer game scores. Computer game designers may also be ignorant about the development status of children, which might complicate the gaming operations for children, and it might be beyond the abilities of some children. As motor development of preschool children is not mature, somatosensory games could be integrated into computer games in the future.

In addition, the computer game play uses keyboard and mouse. Because the physical and cognitive development of preschool children is immature at this age, maneuvering and using keyboard and mouse controls were not easy. Therefore, during the game, researchers can use stickers of different colors to be pasted on the keyboard or mouse, which is convenient for children to identify and operate the game.

In this study, the 4-week computer game intervention had a short-term effect on improving children’s nutritional knowledge and behavior changes. Another study also showed that short-term computer game intervention can increase nutritional knowledge; however, it might not be enough to greatly increase children’s nutritional knowledge and actual eating behavior in the long term [9]. Hence, a follow-up study will be performed to investigate which duration of the computer game intervention would change the long-term eating behaviors of children in the future. Moreover, the future research requires a larger sample size to understand the effectiveness of computer games in nutritional and health education for young children.

### Conclusion

Teaching using a computer game could improve children’s nutritional knowledge. However, the intake frequency of junk food among children in the experimental group showed no significant difference from those in the control group in the posttest.
